# Outcomes after In Utero Myelomeningocele Repair Based on Delivery Location

**DOI:** 10.3390/jcm9113443

**Published:** 2020-10-27

**Authors:** Eric P. Bergh, Kuojen Tsao, Mary T. Austin, Stephen A. Fletcher, Suzanne M. Lopez, Kenneth J. Moise, Anthony Johnson, Ramesha Papanna

**Affiliations:** 1 Departments Obstetrics, Gynecology & Reproductive Sciences and Pediatric Surgery, Division of Maternal-Fetal Medicine, McGovern Medical School at the University of Texas Health Science Center at Houston and Children’s Memorial Hermann Hospital, Houston, TX 77030, USA; eric.p.bergh@uth.tmc.edu (E.P.B.); anthony.johnson@uth.tmc.edu (A.J.); 2 Departments of Pediatric Surgery and Obstetrics, Gynecology & Reproductive Sciences, McGovern Medical School at the University of Texas Health Science Center at Houston and Children’s Memorial Hermann Hospital, Houston, TX 77030, USA; KuoJen.Tsao@uth.tmc.edu (K.T.); Mary.T.Austin@uth.tmc.edu (M.T.A.); 3 Division of Pediatric Neurosurgery, Department of Pediatrics, and Department of Pediatric Surgery, UTHealth the University of Texas McGovern Medical School, Houston, TX 77030, USA; stephen.fletcher@uth.tmc.edu; 4 Department of Pediatrics, McGovern Medical School—UTHealth, Houston, TX 77030, USA; suzanne.m.lopez@uth.tmc.edu; 5 Department of Women’s Health, The University of Texas at Austin, Dell Medical Shool, Austin, TX 77030, USA; Kmoise@austin.utexas.edu

**Keywords:** in utero open fetal myelomeningocele repair, obstetric and neonatal management, delivery location, neonatal outcomes, maternal outcomes, referring physician, spina bifida

## Abstract

Maternal and pediatric delivery outcomes may vary in patients who underwent open fetal myelomeningocele repair and elected to deliver at the fetal center where their fetal intervention was performed versus at the referring physician’s hospital. A prospective cohort study of 88 patients were evaluated following in utero open fetal myelomeningocele repair at a single fetal center between the years 2011–2019. Exclusion criteria included patients that delivered within two weeks of the procedure (*n* = 6), or if a patient was lost to follow-up (*n* = 1). Of 82 patients meeting inclusion criteria, 36 (44%) patients were delivered at the fetal center that performed fetal intervention, and 46 (56%) were delivered locally. Comparative statistics found that with the exception of parity, baseline characteristics and pre-operative variables did not differ between the groups. No differences in oligohydramnios incidence, preterm rupture of membranes, gestational age at delivery or delivery indications were found. Patients who delivered with a referring physician were more likely to be multiparous (*p* = 0.015). With the exception of a longer neonatal intensive care unit (NICU) stay in the fetal center group (median 30.0 vs. 11.0 days, *p* = 0.004), there were no differences in neonatal outcomes, including wound dehiscence, cerebrospinal fluid leakage, patch management, ventricular diversion, or prematurity complications. Therefore, we conclude that it is safe to allow patients to travel home for obstetric and neonatal management after open fetal myelomeningocele repair.

## 1. Introduction

At many centers in the United States, patients who choose to undergo in utero surgery for open fetal myelomeningocele repair are required to relocate to the surgical center for the duration of their pregnancy. This practice likely originates from a historical requirement imposed upon patients randomized to open fetal myelomeningocele repair in the landmark Management of Myelomeningocele Study (MOMS) trial [1]. However, due to geographic variability in access to specialized fetal therapy centers, the financial and social burden of relocating during pregnancy may prohibit some patients from open fetal myelomeningocele repair. In keeping with recommendations by the Maternal–Fetal Management Task Force [2], our practice is to discharge stable patients home after two weeks of outpatient observation in order to continue prenatal care with their referring physician with an anticipated delivery at their local hospital. It is unknown whether the location of subsequent prenatal care and delivery have an influence on maternal and perinatal outcomes. In this study, we compared obstetric and neonatal outcomes in a population of patients who underwent open fetal myelomeningocele repair and were either delivered at the fetal center where fetal intervention was performed or at their referring physicians’ hospital.

## 2. Materials and Methods

This was a retrospective analysis of a prospective cohort of consecutive patients referred for evaluation of fetal myelomeningocele that underwent open fetal repair at The Fetal Center at Children’s Memorial Hermann Hospital in Houston, TX, USA. The study protocol was approved by the institutional review board (IRB) of the University of Texas Health Science Center at Houston (HSC-15-0168) and was conducted between July 2011 and September 2019. Delivery and neonatal outcomes were collected from the medical records obtained from patients’ delivery hospitals.

All patients with suspected fetal myelomeningocele underwent an extensive pre-operative evaluation, which included detailed fetal imaging by ultrasound and MRI, confirmation of a normal karyotype by amniocentesis, and extensive consultation with maternal–fetal medicine specialists, pediatric surgery, pediatric neurosurgery, anesthesiology, neonatology, nursing, and social work. At our institution, candidates for open fetal surgery are selected based on criteria outlined in the MOMS trial [1]. We also considered patients with a pre-pregnancy body mass index (BMI) between 35 and 40 as eligible for prenatal repair under an IRB approved protocol (HSC-MS-15-0795). Patients meeting inclusion criteria are offered prenatal myelomeningocele repair between 19 weeks 0 days and 25 weeks 6 days gestational age via an open laparotomy and hysterotomy technique [1]. Any patient electing to proceed with surgery was asked to relocate to a residence within 30 min of the fetal center for the first two weeks post-discharge from the hospital, after which patients could return home to be managed by their referring physician. While we did not track the method of transportation, patients were free to travel to their home location via air or car. Prior to leaving the fetal center, the patients’ delivery hospitals were screened for adequate maternal, neonatal, and neurosurgical levels of care, and the referring physicians were provided detailed instructions on continued fetal surveillance and obstetric management, including instructions for qualitative assessment of the hysterotomy incision and timing of delivery. If the patient was unable to identify a delivery hospital with an adequate level of neurosurgical care, they were asked to relocate to Houston at approximately 34 weeks of gestation for delivery at the fetal center. Throughout the remainder of gestation, the fetal center’s team maintained close contact with the discharged patient and the referring physician. In cases where a patch was required to close myelomeningocele defects, detailed instructions on proper patch management were provided to neonatology and neurosurgical teams at the patients’ delivery hospitals. Finally, it is our practice and our recommendation to all referring physicians that patients be managed expectantly until cesarean delivery at 37 weeks, unless there are other indications for an earlier delivery.

This was an analysis of all patients who chose to undergo prenatal myelomeningocele repair during the study period, and who subsequently delivered more than two weeks after discharge from the fetal center. The following baseline characteristics were collected: gestational age at the time of evaluation, age, gravidity, parity, body mass index (BMI), race, distance travelled between the fetal center and the delivery hospital, region of the country where the patient’s delivery hospital is located, cervical length, presence of an anterior placenta, type of lesion, level of the lesion, average lateral ventricle size, presence of talipes, presence of lower extremity movement, and the gestational age at surgery. Presence of fetal limb movement was defined as evidence of bilateral full extension and return to flexion at each lower extremity joint, observed on ultrasound. Post-operative maternal variables included gestational age at delivery (weeks), incidence of membrane separation, wound infection, oligohydramnios, chorioamnionitis, indication for delivery, blood transfusion at delivery, and status of the hysterotomy at cesarean section. Post-operative neonatal variables collected from birth until discharge included birthweight (g), incidence of respiratory distress syndrome (RDS), apnea, pneumothorax, patent ductus arteriosus, culture proven sepsis, the need for a patch at prenatal myelomeningocele repair, dehiscence of the prenatal repair, cerebrospinal fluid leakage at birth, placement of a ventriculo-peritoneal diversion shunt, and the length of neonatal intensive care unit (NICU) stay. All neonatal outcomes represent data collected from time of birth until hospital/NICU discharge.

Baseline characteristics and operative outcomes were compared between patient groups. Normality testing of continuous data was performed using the Shapiro–Wilk normality test. Results have been reported as either a percentage or mean with standard deviation or as a median with an interquartile range (IQR). This was followed by significance testing using *t*-test, Kruskal–Wallis, chi-square, or Fisher exact test as appropriate. All analyses were conducted in R version 3.6.0; the “survminer” and “survival” packages were used for survival analysis, and the “geosphere” package was used to compute the great-circle distance between the fetal center and patients’ place of origin [3,4,5,6]. A *p*-value of <0.05 was considered significant.

## 3. Results

A total of 167 patients were referred to the fetal center for evaluation of fetal myelomeningocele during the study period and subsequently delivered. One hundred patients (59.9%) were considered appropriate candidates for open fetal surgery, of which 88 (88%) choose to proceed with prenatal repair. Six patients (6.8%) who delivered within two weeks of the procedure while under initial post-operative observation at the fetal center were excluded from analysis. In total, 82 patients remained for analysis, of which delivery and NICU outcomes were available for 81 patients; one patient was lost-to follow-up. Thirty-six patients (43.9%) delivered at the fetal center, whereas 46 patients (56.1%) travelled home to deliver with their referring physician.

With the exception of parity, no differences were found in any of the variables collected between the fetal center and referring physician groups upon evaluation of baseline and operative characteristics in the study cohort (Table 1). Among the entire cohort (*n* = 82), the average maternal age was 29.1 (±5.01) having an average BMI of 28.0 kg/m^2^ (±4.42). While parity overall was similar between the groups, patients who travelled back to their referring physician were more likely to be multiparous (*p* = 0.015). The majority of patients identified as white (65.9%), and approximately half had an anterior placenta (48.8%). The majority of bony lesions began at L3–L4 (53.7%), and 54.9% of fetuses had a myelomeningocele versus a myeloschisis (45.1%). Median lateral ventricle size was 11.7 mm (IRQ 9.39; 14.2) and 29.3% of fetuses had talipes. Over 85% of fetuses had bilateral hip, knee, and ankle movement. The median gestational age at the time of prenatal surgery was 25.1 weeks (IQR 24.7; 25.6) and the median length of hospital stay after fetal surgery was 4 days for the entire cohort.

**Figure 1:**
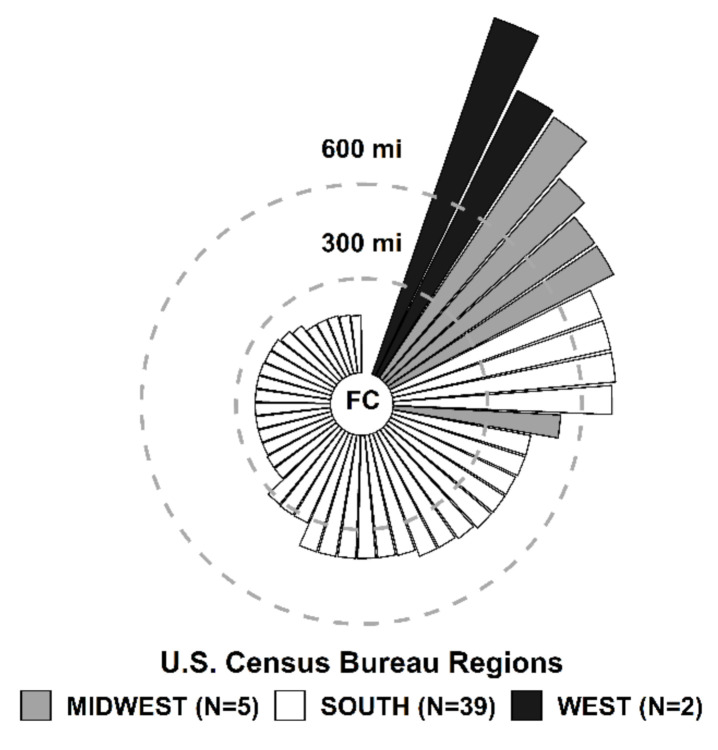
(Miles) from the fetal center (FC) to delivery hospital associated with referring physician in 46 patients who were discharged home 2 weeks after prenatal myelomeningocele (MMC) repair.

Figure 1 shows the distance from the fetal center to the site of the referring physician’s delivery hospital for the 46 patients who travelled home two weeks after prenatal myelomeningocele repair. Referring physician locations are further characterized by their associated U.S. Census Bureau Region; Midwest (*n* = 5, 10.9%), South (*n* = 39, 84.8%), and West (*n* = 2, 4.3%). The majority of patients travelled to the fetal center from Southern states and from distances greater than 300 miles away (*n* = 28, 60.9%).

Postoperative maternal outcomes are presented in Table 2. The status of the hysterotomy at delivery could not be extracted from the chart of a single patient in the referring physician group. There was no difference noted in gestational age at delivery, the incidences of membrane separation, wound infection, oligohydramnios, chorioamnionitis, indications for delivery, blood transfusion at delivery, and the status of the hysterotomy at cesarean section. These data suggest that maternal outcomes at delivery were independent of delivery location.

**Figure 2:**
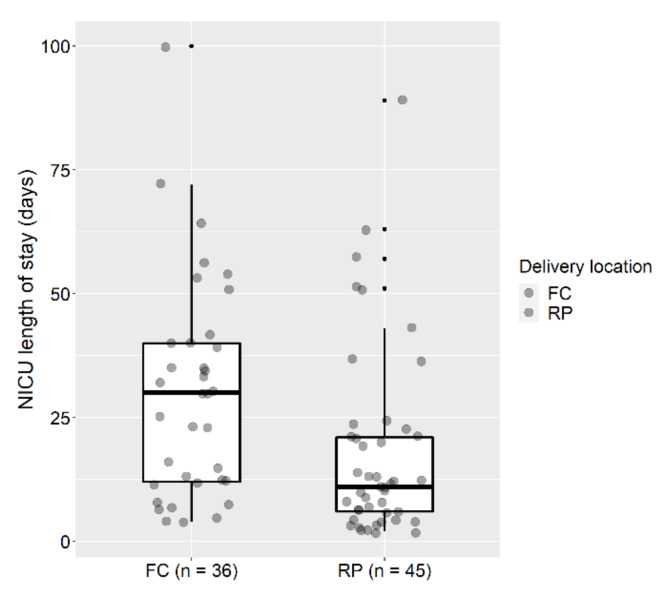
Length of stay (days) versus delivery location in 81 patients who underwent prenatal myelomeningocele repair. FC, fetal center; RP, referring physician.

With the exception of NICU length of stay (*p* = 0.004), there were no differences found in any neonatal outcomes, including gestational age at delivery, birth weight, respiratory distress syndrome, apnea, pneumothorax, patent ductus arteriosus, necrotizing enterocolitis, placement of skin and/or dura patch at surgery, rates of patch dehiscence, or cerebrospinal fluid leakage at birth or in ventriculo-peritoneal shunt or endoscopic third ventriculostomy procedure rates (Table 3). More importantly, there were no culture-proven sepsis or perinatal deaths in either group. As mentioned, NICU length of stay was significantly longer in the fetal center group compared with the referring physician group (30 days (IQR 12.0; 40.0) versus 11 days (IQR 6.0; 23.0), *p* = 0.004). We found consistently greater lengths of NICU stay at the fetal center independent of whether the babies were discharged at <2 weeks, 2–4 weeks or >1 month from delivery (*p* = 0.024). The distribution of NICU stay for each group is graphically presented in Figure 2.

**Figure 3:**
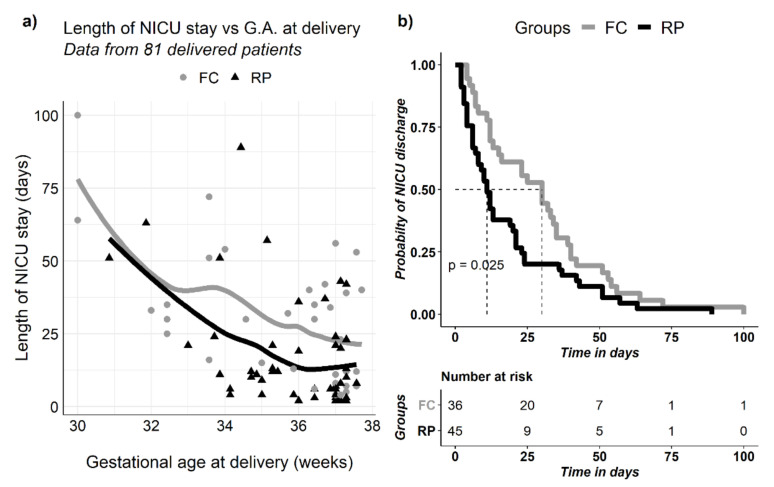
NICU length of stay stratified by delivery location groups: (**a**) NICU length of stay (days) versus gestational age at delivery in 81 patients who underwent prenatal MMC repair. Curves represent LOESS fit for distribution by delivery location. (**b**) Kaplan–Meir analysis of NICU length of stay (days) in 81 patients who underwent prenatal MMC repair. NICU, neonatal intensive care unit; G.A., gestational age; FC, fetal center; RP, referring physician; MMC myelomeningocele, LOESS, local polynomial regression. *p*-value represents log rank test.

Figure 3a illustrates the total length of NICU stay versus gestational age at delivery for each group. Local polynomial regression curves for each distribution reveal a similar trend at both delivery sites, a reduction in hospitalization duration with increasing gestational age at delivery. Notably, prolonged hospital stay was seen in neonates born at the fetal center when compared to the referring physician group. (Figure 3b; log rank test *p* = 0.025). The main difference appears to be among children discharged less than two weeks after birth and greater than one month after birth (Table 3).

## 4. Discussion

In this study, we have demonstrated that delivery location does not appear to adversely affect maternal outcomes in women who undergo open fetal myelomeningocele repair. Neonatal outcomes appear similar with the exception of a longer NICU stay in the fetal center group. Although many centers routinely allow patients to return home for post-operative care and delivery, this study is the first to substantiate the safety of this practice.

For patients who receive a prenatal diagnosis of fetal myelomeningocele and who are appropriate surgical candidates, prenatal surgery is an opportunity to potentially improve the quality of life of their unborn child. Specifically, open fetal myelomeningocele repair is associated with reduced incidence of shunt placement and hindbrain herniation at 12 months, and increased independent walking at 30 months, compared with children who undergo postnatal repair [1,7,8]. However, the benefits of fetal surgery may not be realized by all if barriers to advanced medical practice prevents patients from pursuing specialized care.

The factors preventing patients from seeking fetal therapy have not been previously identified. However, questions surrounding the financial and social burden of travel and relocation may weigh heavily upon patients already struggling with the implications of caring for a child with a rare and possibly life-limiting diagnosis. In this cohort, patients who travelled home to their referring physician were more likely to be multiparous. It may be that patients with additional children at home are more likely to seek care at a distant fetal center if they are given an opportunity to return home to their families for the duration of their pregnancy.

We have previously published on the safety of traveling long distances to obtain care for twin-twin transfusion syndrome via minimally invasive, fetoscopic surgery [9]. These patients routinely travel upwards of 500 miles for fetal surgery and receive post-operative and delivery care by their referring physician without adversely affecting gestational age at delivery or neonatal survival. While a direct comparison with patients who undergo open fetal surgery is impossible, the majority of patients in this study who returned to a referring physician travelled greater than 300 miles, presumably via both air and car, although this data was not routinely collected. This is notable given the increased morbidity associated with laparotomy and hysterotomy performed at time of open fetal myelomeningocele repair.

In this cohort of patients undergoing open fetal myelomeningocele repair, there were significant differences in the duration of NICU stay between delivery groups; this occurred despite similar neonatal outcomes including gestational age at birth, birthweight, patch requirement, ventricular diversion procedure, and other complications of prematurity. A possible explanation may be differences in practice management among NICU and neurosurgical teams, with ours favoring a more conservative approach with longer periods of in-house observation. Another possible explanation would be a higher incidence of hospital readmission in the referring physician group, although this is not a variable that we routinely collect. However, the finding that NICU length of stay decreases with increasing GA at delivery in both groups is reassuring.

Contemporary cohorts of patients undergoing open prenatal myelomeningocele repair have reported similar prolonged neonatal lengths of stay. Riddle et al. reported a median (IQR) length of stay of 53 (15; 85) days amongst 23 patients who underwent prenatal repair, although the mean gestational age at delivery in this cohort was 30.8 (±4.4) weeks [10]. Cools et al. reported a median (range) length of stay of 25.5 (5.5–41.5) days in a cohort of 24 patients delivered at 32.1 (±3.1) weeks [11]. Among 91 patients who underwent prenatal repair in The Management of Myelomeningocele Study (MOMS) and delivered at 34.1 (±3.0) weeks, length of NICU stay was 19.2 (±19.3) days [12]. Clearly, gestational age at birth is a significant determinant of NICU length of stay, although this cannot be the only contributing factor as the median (IQR) gestational age at delivery in our cohort of patients delivered at the Fetal Center was 36.6 (33.9; 37.2) weeks. In addition to unanticipated neonatal complications, it is likely that regional and individual practices may drive some of the differences amongst centers.

A major limitation of this study is the lack of socioeconomic status information and the related impact on long-term pediatric outcomes between the groups. Previous studies have suggested that the benefits associated with prenatal repair, such as an increased mental ability from decreased shunting and mobility independence, are the main determinants of myelomeningocele children’s qualities of life [13]. However, in spite of the benefits associated with prenatal repair, there is evidence to suggest that socioeconomic factors, such as family income and education, may also contribute substantially to the quality of life of myelomeningocele children [14,15]. We cannot eliminate the possible bias that one’s proximity to a major medical center in a large metropolitan area may facilitate the coordination of complex life-long care and potentially an improved quality of life and long-term outcomes. Additionally, the majority of our patients delivered in the Southern U.S. Regional variations (both nationally and internationally) may limit the applicability of our results to other populations of patients undergoing open fetal myelomeningocele repair.

Finally, there are likely emotional as well as physiological benefits to experiencing post-surgical recovery in a location where family support is greatest. In general surgery literature, patients with elevated pre-operative cortisol levels when in combination with a perceived family support deficit are far more likely to suffer post-operative complications, such as fever, excessive pain, and prolonged hospital stays [16]. To our knowledge, no studies to date have been reported in fetal surgery literature, however it is known that stress during pregnancy is a known risk factor for poor reproductive outcomes, including increased rates of preterm birth and low birthweight [17]. In this study, we were unable to capture the perceived psychological benefits of recovery in the referring physician’s locale, however this could be an area of future research.

In summary, our results suggest that a policy of allowing patients to return to the site of their referring physician after an observation period of two weeks post-prenatal myelomeningocele repair does not affect maternal or neonatal outcomes. Given the extreme financial, social, and emotional burden associated with relocation during pregnancy, this practice appears to be safe to increase access to care for all patients, regardless of their proximity to a major fetal center.

## 5. Conclusions

The location of delivery after open fetal myelomeningocele repair does not appear to adversely affect maternal or neonatal outcomes. Regional variations in care may contribute to differences in post-natal management, such as the duration of NICU length of stay. However, it appears safe to allow patients to travel home for obstetric and neonatal care after open fetal myelomeningocele repair.

## Figures and Tables

**Table 1:** Baseline and operative characteristics of the study population (*n* = 82).

Characteristic	Entire Cohort	FC	RP	*p* *
	*n* = 83	*n* = 36	*n* = 47	
G.A. at evaluation (w)	23.1 (22.1; 24.0)	23.0 (21.8; 23.7)	23.1 (22.4; 24.1)	0.262
Maternal age (years)	29.1 (±5.01)	28.6 (±4.9)	29.6 (±5.1)	0.369
BMI (kg/m^2^)	28.0 (±4.42)	28.3 (±4.64)	27.8 (±4.28)	0.625
Parity	1.00 (0.00; 2.00)	1.00 (0.00; 2.00)	1.00 (0.00; 2.00)	0.078
Parity (grouped)				0.015
Nulliparous	26 (31.7%)	17 (47.2%)	9 (19.6%)	
Multiparous	56 (68.3%)	19 (52.8%)	37 (80.4%)	
Race				0.290
White	54 (65.9)	20 (55.6)	34 (73.9)	
African American	4 (4.88)	2 (5.56)	2 (4.35)	
Hispanic or Latino	22 (26.8)	13 (36.1)	9 (19.6)	
Asian	2 (2.44)	1 (2.78)	1 (2.17)	
Cervical length (mm)	42.0 (36.0; 47.0)	39.5 (33.8; 45.1)	42.9 (37.3; 47.8)	
Anterior placenta	40 (48.8)	18 (50.0)	22 (47.8)	1.00
Type of lesion:				1.00
MMC	45 (54.9)	20 (55.6)	25 (54.3)	
Myeloschisis	37 (45.1)	16 (44.4)	21 (45.7)	
Level of the lesion				0.536
L1–L2	20 (24.4)	9 (25.0)	11 (23.9)	
L3–L4	44 (53.7)	17 (47.2)	27 (58.7)	
L5–S1	14 (17.1)	7 (19.4)	7 (15.2)	
Thoracic	4 (4.88)	3 (8.33)	1 (2.17)	
Lateral ventricle (mm) ^†^	11.7 (9.39; 14.2)	11.7 (9.24; 15.6)	11.7 (9.50; 13.6)	0.698
Presence of talipes:	24 (29.3)	11 (30.6)	13 (28.3)	1.00
Presence of limb movement: ^‡^				
Hips	80 (97.6)	36 (100)	44 (95.7)	0.501
Knees	77 (93.9)	34 (94.4)	43 (93.5)	1.00
Ankles	68 (85.0)	30 (85.7)	38 (84.4)	1.00
G.A. at prenatal surgery (wks)	25.1 (24.7; 25.6)	25.1 (24.9; 25.5)	25.1 (24.7; 25.6)	0.862
Post-operative LOS (days)	4.00 (4.00; 4.00)	4.00 (4.00; 5.00)	4.00 (4.00; 4.00)	0.120

FC, fetal center; RP, referring physician; G.A., gestational age; BMI, body mass index; MMC, myelomeningocele; LOS, inpatient length of stay post fetal surgery. Data are mean ± SD, median(IQR) or *n*(%) * Comparisons between FC and RP; *t*-test, Kruskal–Wallis, Chi-square or Fisher’s Exact test used as appropriate † Represented as the mean of the left and right lateral ventricles measured across the atria. ‡ Defined as the presence of full extension and flexion of bilateral joints.

**Table 2:** Maternal delivery outcomes. *

Parameter	FC	RP	*p* **
	*n* = 36	*n* = 45 ^†^	
G.A. at delivery (w)	36.6 (33.9; 37.2)	36.0 (34.9; 37.0)	0.786
Membrane separation	3 (8.3)	0	0.084
Wound infection	2 (5.6)	1 (2.2)	0.582
Oligohydramnios	6 (16.7)	3 (6.67)	0.176
Chorioamnionitis	0	2 (4.44)	0.500
Indication for delivery: ^‡^			
Abruption	0	2 (4.4)	0.500
Premature rupture of membranes	8 (22.2)	7 (15.6)	0.631
Preterm labor or contractions	15 (41.7)	13 (28.9)	0.334
Scheduled	19 (52.8)	23 (51.1)	1.000
Status of hysterotomy at delivery: ^§^			0.881
Intact	30 (83.3)	35 (79.5)	
Thin	5 (13.9)	8 (18.2)	
Focal or area of dehiscence	1 (2.78)	1 (2.27)	

FC, fetal center; RP, referring physician; G.A., gestational age; Data are median (IQR) or *n* (%) * There were no instances of blood transfusion in either group. ** Comparisons between FC and RP; Kruskal–Wallis, Chi-square or Fisher’s Exact test used as appropriate ^†^ One lost-to follow-up patient was excluded from the initial cohort of 46 patients ^‡^ Percentage may not total 100% as some patients had multiple delivery indications. ^§^ Data for one patient in the RP group was not available for analysis.

**Table 3:** Neonatal outcomes. *

Parameter	FC	RP	*p*
	*n* = 36	*n* = 45 ^†^	
G.A. at delivery (w)	36.6 (33.9; 37.2)	36.0 (34.9; 37.0)	0.786
Birth weight (g)	2604 (±567)	2686 (±479)	0.492
Any respiratory distress ^‡^	4 (11.1)	14 (31.1)	0.060
Respiratory distress syndrome ^§^	1 (2.78)	1 (2.22)	1.000
Apnea	8 (22.2)	10 (22.2)	
Pneumothorax	0	1 (2.22)	
Patent ductus arteriosus	1 (2.78)	0	0.444
Necrotizing enterocolitis	1 (2.78%)	0	0.444
Need for a patch at prenatal MMC surgery	16 (44.4)	15 (33.3)	0.428
Patch location ^¶^			0.654
Skin only	12 (75.0)	13 (86.7)	
Skin and dura	4 (25.0)	2 (13.3)	
Dehiscence repair	6 (16.7)	3 (6.67)	0.176
CSF leakage at birth	1 (2.78)	2 (4.44)	1.000
VP shunt placement	7 (19.4)	7 (15.6)	0.870
ETV procedure	1 (2.7)	1 (2.2)	1.000
Length of NICU stay (days)	30.0 (12.0; 40.0)	11.0 (6.0; 23.0)	0.004
NICU stay (grouped)			0.024
<2 weeks	12 (33.3)	28 (62.2)	
2–4 weeks	8 (22.2)	8 (17.8)	
>1 month	16 (44.4)	9 (20.0)	

FC, fetal center; RP, referring physician; G.A., gestational age; MMC, myelomeningocele; CSF, cerebrospinal fluid; VP, ventriculo-peritoneal; ETV, endoscopic third ventriculostomy; NICU, neonatal intensive care unit. Data are mean ± SD, median (IQR) or *n*(%) * There were no instances of sepsis or perinatal death in either group. ** Comparisons between FC and RP; Kruskal–Wallis, Chi-square or Fisher’s Exact test used as appropriate† One lost-to follow-up patient was excluded from the initial cohort of 46 patients. ^‡^ Any documentation of respiratory distress by the NICU provider, irrespective of FiO_2_. ^§^ Defined as documentation of ≥24 h of ≥0.4% FiO_2_ at birth ^¶^ Patch material selected at the surgeon’s discretion; Skin patch material included: Durepair^®^ (*n* = 25), DuraGen^®^ (*n* = 1), AlloDerm^®^ (*n* = 1), AmnioGuard^®^ (*n* = 4); Dura patch material included: Durepair^®^ (*n* = 4), AlloDerm^®^ (*n* = 1), AmnioGuard^®^ (*n* = 1).

## References

[1] Adzick N.S., Thom E.A., Spong C.Y., Brock J.W., Burrows P.K., Johnson M.P., Lori M.D., Howell J., Jody M.S., Farrell A. (2011). A randomized trial of prenatal versus postnatal repair of myelomeningocele. N. Engl. J. Med..

[2] Cohen A.R., Couto J., Cummings J.J., Johnson A., Joseph G., Kaufman B.A., Litman R.S., Kathryn Menard M., Moldenhauer J.S., Kevin C. (2014). Position statement on fetal myelomeningocele repair. Am. J. Obstet. Gynecol..

[3] R Core Team (2019). R: A Language and Environment for Statistical Computing.

[4] Kassambara A., Kosinski M., Biecek P. (2020). Survminer: DrawingSurvival Curves Using ‘ggplot2’. https://CRAN.R-project.org/package=survminer.

[5] Therneau T.M. (2015). A Package for Survival Analysis in S. https://CRAN.R-project.org/package=survival.

[6] Hijmans R.J. (2017). Geosphere: Spherical Trigonometry. https://CRAN.R-project.org/package=geosphere:https://CRAN.R-project.org/package=geosphere.

[7] Tulipan N., Wellons J.C., Thom E.A., Gupta N., Sutton L.N., Burrows P.K., Farmer D.L., Walsh W.F., Johnson M.P., Rand L. (2015). Prenatal surgery for myelomeningocele and the need for cerebrospinal fluid shunt placement. J. Neurosurg. Pediatr..

[8] Farmer D.L., Thom E.A., Brock J.W., Burrows P.K., Johnson M.P., Howell L.J., Farrell J.A., Gupta N., Adzick N.S., Management of Myelomeningocele Study Investigators (2018). The Management of Myelomeningocele Study: Full cohort 30-month pediatric outcomes. Am. J. Obstet. Gynecol..

[9] Bergh E.P., Donepudi R., Bell C.S., Moise K.J., Johnson A., Papanna R. (2020). Distance Traveled to a Fetal Center and Pregnancy Outcomes in Twin-Twin Transfusion Syndrome. Fetal Diagn. Ther..

[10] Riddle S., Huddle R., Lim F.Y., Stevenson C., Dean K., Sparling K., Fenchel M., Schibler K. (2019). Morbidity and cost burden of prenatal myelomeningocele repair. J. Matern. Fetal Neonatal Med..

[11] Cools M., Northam W., Goodnight W., Mulvaney G., Elton S., Quinsey C. (2019). Thirty-day medical and surgical readmission following prenatal versus postnatal myelomeningocele repair. Neurosurg. Focus..

[12] Rintoul N.E., Keller R.L., Walsh W.F., Burrows P.K., Thom E.A., Kallan M.J., Howell L.J., Adzick N.S., for the MOMS Investigators (2020). The Management of Myelomeningocele Study: Short-Term Neonatal Outcomes. Fetal Diagn. Ther..

[13] Schoenmakers M.A.G.C., Uiterwaal C.S.P.M., Gulmans V.A.M., Gooskens R.H.J.M., Helders P.J.M. (2005). Determinants of functional independence and quality of life in children with spina bifida. Clin. Rehabil..

[14] Kulkarni A.V., Cochrane D.D., McNeely P.D., Shams I. (2008). Medical, Social, and Economic Factors Associated with Health-Related Quality of Life in Canadian Children with Hydrocephalus. J. Pediatr..

[15] Tezcan S., Şimşek T.T. (2013). Comparison of health-related quality of life between children with cerebral palsy and spina bifida. Res. Dev. Disabil..

[16] Cardoso-Moreno M.J., Tomás-Aragones L. (2016). The influence of perceived family support on post surgery recovery. Psychol. Health Med..

[17] Katz V.L. (2012). Work and Work-related Stress in Pregnancy. Clin. Obstet. Gynecol..

